# The Effect of Venoactive Drug Therapy on the Development and Severity of Post-Embolization Syndrome in Endovascular Interventions on the Gonadal Veins

**DOI:** 10.3390/jpm11060521

**Published:** 2021-06-07

**Authors:** Sergey Gennadievich Gavrilov, Gennady Vladimirovich Krasavin, Nadezhda Yurievna Mishakina, Oksana Igorevna Efremova, Igor Anatolievich Zolotukhin

**Affiliations:** Department of Fundamental and Applied Research in Surgery, Pirogov Russian National Research Medical University, 10/5 Leninsky Prospect, 119049 Moscow, Russia; gavriloffsg@mail.ru (S.G.G.); gkrasavin@mail.ru (G.V.K.); mishakina.78@mail.ru (N.Y.M.); lpl2@yandex.ru (O.I.E.)

**Keywords:** pelvic congestion syndrome, endovascular embolization of the gonadal veins, post-embolization syndrome, venoactive drug

## Abstract

**Objective.** To evaluate the incidence of post-embolization syndrome (PES) and the effect of venoactive therapy on its development, severity, and duration after endovascular embolization of gonadal veins (EEGV) with coils in patients with pelvic congestion syndrome (PCS). **Materials and Methods.** We analyzed the outcomes of EEGV with coils in 70 female patients who received (*n* = 38; group 1) or did not receive (*n* = 32; group 2) treatment with a venoactive drug (VAD) before and after the procedure. Assessments of the EEGV efficacy and for possible signs of PES were done on days 1, 5, 10, 15, 20, and 30 after the intervention. All patients underwent transvaginal and transabdominal duplex ultrasound scanning (DUS) after EEGV. In addition, patients with PES were examined using the computed tomography of the pelvic veins in the postprocedural period. **Results.** Technical success of EEGV was achieved in 100% of patients. Pelvic venous pain (PVP) reduction after EEGV was observed in 77.1% of patients. The PES was diagnosed in 18.6% of cases (10.5% in group 1 vs. 28.1% in group 2, *p* > 0.05). In three patients of group 1, the protrusion of coils was suspected and eventually verified during the resection of the left gonadal vein with coils. The group 1 patients had less severe post-embolization pain (6.2 ± 0.4 vs. 7.8 ± 0.3 scores in group 2; *p* = 0.009) and three times shorter duration of PES (5.0 ± 1.2 vs. 16.2 ± 2.7 days; *p* = 0.003). No significant differences in the diameters of gonadal veins, side of embolization, and number of coils were revealed between patients with and without PES. The rate of parametrium vein thrombosis was found to be significantly higher in patients with PES than in those without PES (30.7% vs. 18.5%, respectively; *p* < 0.05). **Conclusion.** The PES is a frequent complication of EEGV with coils and occurs in 18.6% of patients. Venoactive treatment does not effect the incidence of this complication but reduces the PES severity and duration.

## 1. Introduction

Endovascular embolization of the gonadal veins (EEGV) is a pathogenetic and minimally invasive method for eliminating pelvic venous reflux in patients with pelvic congestion syndrome (PCS) [1,2,3,4]. Despite its high efficacy in relieving PCS, a number of researchers reported persistence or even an increase of pain after the procedure [5,6,7]. This condition is known as post-embolization syndrome (PES). After implantation of metal coils into ovarian veins, some patients experience worsening of pelvic pain, occurrence of pain along the embolized vessel, and a low-grade fever. This can be explained by the development of aseptic inflammation in the venous wall and/or by the patient’s hypersensitivity to metals and alloys of which coils are made. PES usually lasts from three days to one month, depending on the patient’s characteristics, severity of pelvic vein dilation, and the type of embolization agents used [8]. The treatment of PES is medical and is aimed at syndrome relief and faster rehabilitation of the patient. Venoactive drugs (VADs) are successfully used to relieve symptoms of chronic venous disease (CVD) and PCS and results in pain relief in most patients who have no indications for surgical or endovascular treatment [9,10]. Pathophysiological mechanisms underlying the analgesic action of VADs are thought to be the effect of VAD on leukocyte adhesion, a decrease in venous wall inflammation, and a decrease in the synthesis of algogens.

The aim of the present study was to evaluate the incidence of PES and the effect of treatment with VAD on its development, severity, and duration in patients with PCS who underwent ovarian vein embolization with coils.

## 2. Methods

A total of 935 female patients were diagnosed with PCS at the Department of Fundamental and Applied Research in Surgery in the period from 2012 to 2020, of whom 203 underwent surgery and 732 received only medical treatment. Among the operated patients, 92 underwent open extraperitoneal resection of the gonadal veins, 29 underwent endoscopic resection of the gonadal veins, and 82 underwent EEGV with coils. This prospective cohort study included 70 consecutive female patients who had undergone EEGV in 2012–2020. The study was approved by the local ethics committee of the Pirogov Russian National Research Medical University (Protocol No 63–112). All patients provided written informed consent before participation in the study. The research was performed in the frame of a clinical study registered at Clinicaltrials.gov (NCT03921788). All methods were carried out in accordance with the relevant guidelines and regulations.

*Inclusion criteria* were age 18 to 50 years; presence of PCS symptoms (pelvic venous pain, heaviness in the hypogastric region, dyspareunia); dilation of gonadal, parametrial or uterine veins with reflux duration of more than 1 s, according to transabdominal and transvaginal duplex ultrasound scanning (TADUS, TVDUS); diameter of gonadal veins not greater than 10 mm; and patient’s written informed consent.

*Exclusion criteria* were pregnancy; history of allergic reactions to contrast media or metals; and presence of nutcracker and May–Thurner syndromes (according to multislice computed tomography (MSCT) and multiplanar venography (MPV) of the pelvic veins).

**Figure 1:**
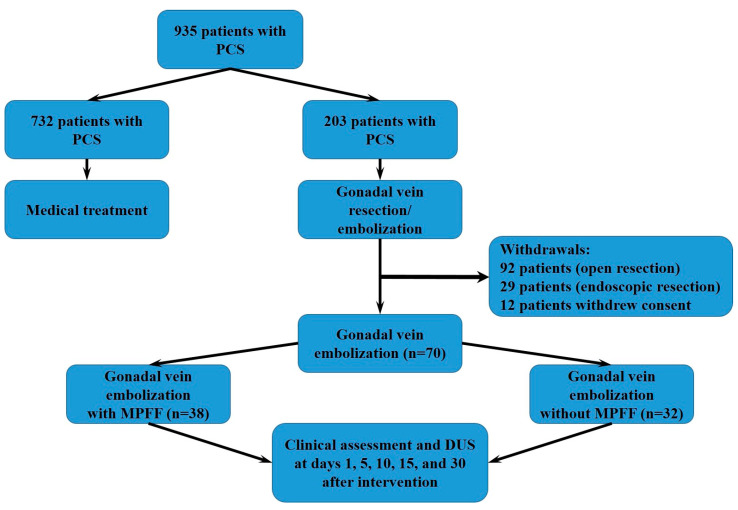
The study flowchart.

Eligible patients were allocated to two groups. Group 1 consisted of 38 patients who underwent EEVG in 2016–2020 and received VAD (micronized purified flavonoid fraction, MPFF) at a dose of 1000 mg daily for 1 month before and 1 month after EEGV. Group 2 consisted of 32 patients who underwent EEVG in 2012–2015 and did not receive VAD. All patients of both groups received 75 mg of diclofenac intramuscularly (i.m.) on the next day after embolization. The study flowchart is presented in Figure 1.

In patients diagnosed with PES, diclofenac 75 mg/day i.m. was administered for 3 to 7 days. In patients with confirmed pelvic vein thrombosis, anticoagulant therapy (enoxaparin 1 mg/kg BID for 7 days, then rivaroxaban 20 mg OD for 1 month) was prescribed.

The EEGV-related pelvic venous pain (PVP) and PES symptoms were evaluated on days 1, 5, 10, 15, 20, and 30 after intervention. In addition to physical examination and evaluation of PVP severity by visual analogue scale (VAS), all patients underwent TADUS and TVDUS of pelvic veins on the next day after EEGV. If post-embolization pain persisted for 3 days, MSCT of pelvic veins was performed in order to exclude or confirm perforation of gonadal veins by coils.

All the patients were informed of the goals of the procedure, its details, and possible complications.

Dilation of the gonadal veins of more than 10 mm with a reflux on DUS in patients who had no May–Thurner and/or nutcracker and/or ovarian veins syndromes according to DUS or pelvic venography and renal venography, high requirements for aesthetic results of the procedure were considered as indications to gonadal veins embolization.

Open or endoscopic resection of GV were conducted in patients with refluxing GVs of 10 mm and more with no May–Thurner and/or nutcracker and/or ovarian veins syndromes if they had an allergic reaction to metals or if there were no coils and catheters available due to logistic reasons. GV’s resection was performed also in patients who refused to undergo EEGV.

Venoactive drug therapy alone was prescribed in patients with dilation and reflux of parametric and uterine veins and no GVs dilation.

### Endovascular Embolization of Gonadal Veins with Coils

The EEGV was performed under local anesthesia with 5.0–10.0 mL of 0.5% lidocaine solution. The left gonadal vein (LGV) embolization was performed using the transfemoral approach, while the right gonadal vein (RGV) embolization or bilateral occlusion of GV was performed using the transjugular approach. The 5F multipurpose angiographic catheters (Cordis; Santa Clara, CA, USA), and standard moving core J 0.035 ” guidewire and an angled hydrophilic wire (Radiofocus; Terumo Corp., Tokyo, Japan) were used. For the GV occlusion, the pushable 0.035 ” standard stainless-steel coils (Gianturco; William Cook, Bjaeverskov, Denmark) and 0.035 ” coils made of Inconel with interwoven long collagen fibrils (MReye; Cook Medical Inc., Bloomington, IN, USA) were used. The diameter of coils was 8–12 mm, and the length was 10–20 cm. When selecting the coil size, the principle of 20–30% coil oversizing relative to the GV diameter was used. After embolization, control ovarian venography was performed.

Statistical analysis was carried out using the MS Excel Statistica 6.0 software and VassarStats online calculator (open-source project). Data are presented as absolute and relative values with mean (M) and standard deviation (σ). Fisher’s exact test was used to compare qualitative variables, and Student’s *t*-test or Mann–Whitney U test were used to compare quantitative variables where appropriate. Differences were considered statistically significant at *p* value < 0.05.

## 3. Results

Baseline characteristics of patients and technical features of EEGV are presented in Table 1. No statistically significant differences between the groups were observed at baseline.

**Figure 2:**
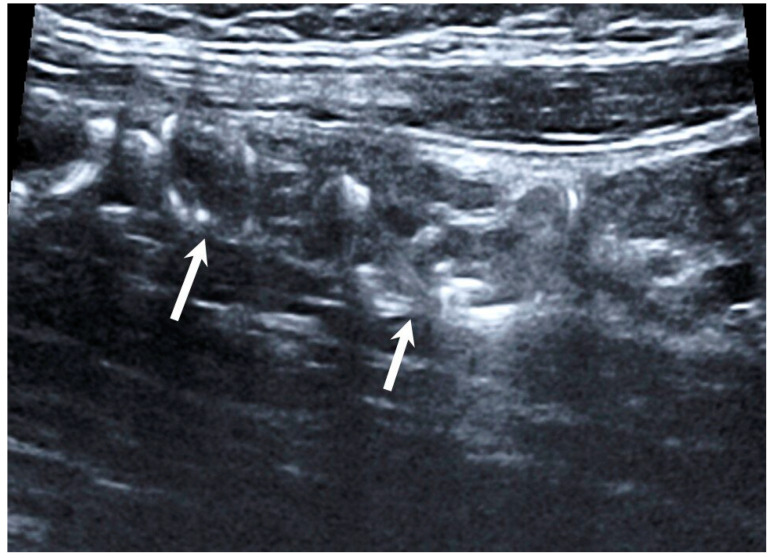
Duplex ultrasound scanning. Hyperechoic structures (coils) in the lumen of the gonadal vein (arrows).

Technical success of EEGV, i.e., elimination of gonadal veins reflux, was achieved in 100% of patients. No complications were observed during the intervention. According to TVDUS, all 70 patients had no blood flow through the occluded gonadal vein segments (Figure 2).

The PVP relief was observed in most patients (77.1%) on day 1 after EEGV regardless of treatment with VAD. PES was diagnosed in 22.9% of total cases, and coil protrusion was detected in 4.3% of patients.

In patients with PES, characteristics of pain were drastically different from the ones for PCS-related pelvic pain, making it easy to distinguish these two conditions. Patients described PVP before EEGV as persistent, dull, aching pain located in the pelvic region and/or in the right or left flanks of the abdomen, often with irradiation to the lower extremities, which aggravated with static and physical loads and did not respond to diclofenac, while women with post-embolization pain described pain as persistent, acute, burning, not related to any load, and was responsive to diclofenac (pain relief within 20–30 min and recurrence in 1–2 h later). Results of comparison of two groups are presented in Table 2.

### 3.1. Group 1

The PVP reduction on day 1 after EEGV was reported by 31 (81.6%) out of 38 patients (mean reduction from 7.8 ± 1.2 to 4.3 ± 1.7 VAS scores). These patients reported further progressive reduction in PVP severity to 1.8 ± 0.6 scores by day 5 with complete elimination of pain on day 10 in 31 patients.

PES was diagnosed in 7 (18.4%) patients who had PVP of 6 to 9 scores (mean 7.3 ± 1.2 scores) prior to EEGV. According to the MSCT data, in these 7 patients, the embolized veins were not contrasted, and no extravasation of contrast medium in the retroperitoneal space was detected. Three patients with low body mass index (BMI) (mean 17.6 ± 0.5 kg/m^2^), in whom PES persisted for more than 30 days after the left-sided EEGV, had pain of 8.6 ± 0.5 VAS scores. This pain only slightly decreased after diclofenac administration. Such a long-term, persistent, intensive pain could be explained by coils’ protrusion that might have led to contact of the coil whorls with the left genitofemoral nerve. Due to persistent pain syndrome, these 3 patients had undergone laparoscopic resection of the left gonadal vein with implanted coils in average 37.6 ± 2.3 days after EEGV. Pain resolved completely at 7 days after resection.

**Figure 3:**
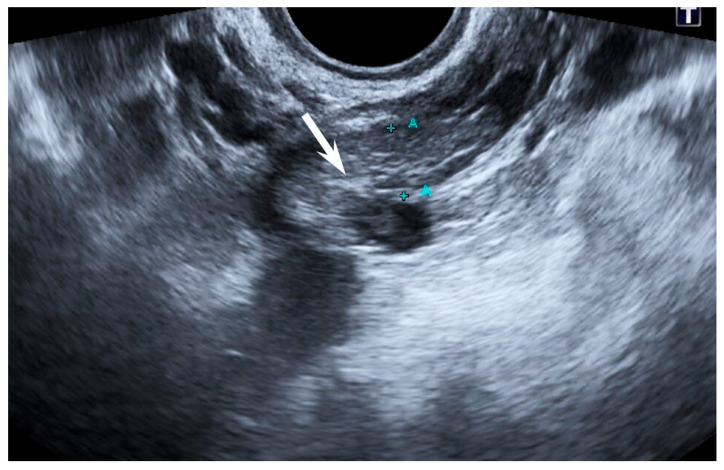
Duplex ultrasound scanning. Blood clot in the lumen of a parametrial vein (arrow).

In the other 4 patients with PES from group 1, mean post-embolization pain score was 6.2 ± 0.4. Three of them underwent left-sided EEGV, and one underwent bilateral EEGV. In these patients, the treatment with anti-inflammatory agents and VAD was associated with a progressive reduction in PES starting from day 3 after EEGV, with reduction to 3.1 ± 0.5 by day 5 and complete resolving by day 10 in all 4 patients. In one patient after bilateral EEVG, thrombosis of parametrial veins was diagnosed on the next day after intervention (Figure 3). These patients received anticoagulant treatment.

### 3.2. Group 2

**Figure 4:**
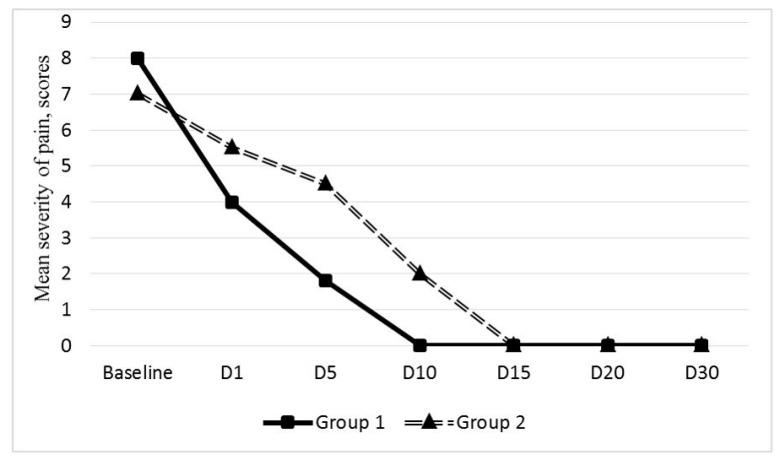
Changes in the mean PVP measured by VAS after the endovascular embolization of gonadal veins in the groups 1 (*n* = 38) and 2 (*n* = 32).

The PVP relief on day 1 after EEVG was observed in 23 (71.8%) out of 32 patients (mean reduction from 6.8 ± 0.3 to 5.5 ± 0.4 VAS scores). The PVP reduction after endovascular intervention was progressive; however, its severity in these 21 patients remained higher than in group 1 patients (Figure 4).

The pain score was 4.4 ± 0.2 on day 5 after EEGV, 1.8 ± 0.4 on day 10, and the complete resolution of PVP was achieved only by day 15.

PES was diagnosed in 9 (28.1%) out of 32 patients. Eight patients underwent left-sided EEGV, and one underwent bilateral EEGV. Mean post-embolization pain score was 7.8 ± 0.3. According to the MSCT data, embolized veins were not contrasted in these 9 patients, and no signs of contrast agent extravasation were detected. Only diclofenac at a dose of 75 mg was used for PES relief.

**Figure 5:**
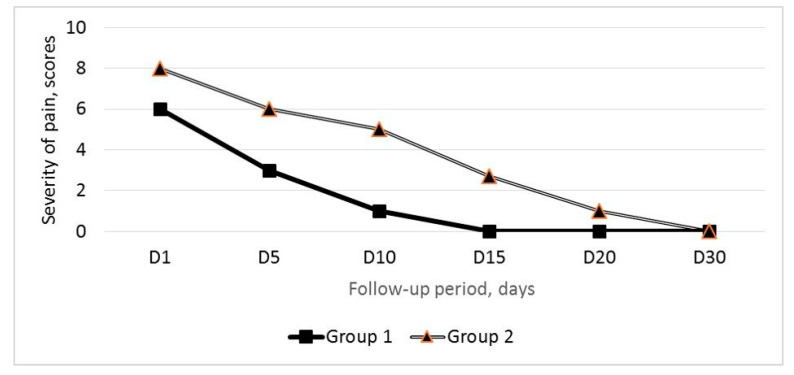
Changes in the mean post-embolization pain score measured by VAS after the endovascular embolization of gonadal veins in the groups 1(*n* = 38) and 2 (*n* = 32).

Mean PES duration in group 2 was 16.2 ± 2.7 days. Despite the fact that post-embolization pain in the pelvic area or in the left flank of the abdomen decreased by day 5 after EEGV to 6.2 ± 0.2 scores, in 5 patients, pain persisted up to day 20 after the procedure (Figure 5).

Parametrial veins thrombosis was diagnosed in 3 patients, of whom 2 underwent left-sided EEGV, and one underwent bilateral EEGV. These patients received anticoagulant therapy.

Comparison between subgroups with and without PES development is presented in Table 3.

Patients with PES had a significantly lower BMI. No significant differences in diameters of the gonadal veins, side of embolization, number of coils used, and rate of parametrial veins thrombosis were found between patients with and without PES. At baseline, patients had similar PVP pain scores; however, after embolization, patients with PES experienced worsening of pain, while patients without PES reported decrease in pain.

Hyperthermia level did not differ significantly between the groups. Body temperature increased to 37.2–37.7 °C during the first week after the procedure. In all 16 patients with PES, complete blood count showed a moderate leukocytosis (9.5–10 × 10^9^/L) in the first days after EEGV. The white blood cell count returned to normal in all patients with PES by day 5 after EEGV. No abnormalities in urine tests and biochemical blood tests were observed.

Mean time to return to work in patients who were treated with MPFF and those who were not was 11 and 25 days, respectively. Three patients who underwent EEGV returned to work on 8th day after procedure.

## 4. Discussion

The EEGV with coils is an effective option for treating PCS [1,2,3,4]. According to various authors [11,12,13,14], this technique provides pelvic pain relief in 53% to 95% of patients with PCS [11,12,13,14]. Complete elimination of blood reflux in the gonadal veins, minimal trauma, and a high cosmetic result, along with the possibility of performing the intervention under local anesthesia, are obvious advantages of EEGV. At the same time, this endovascular technique has some inherent limitations, such as the risk of migration or protrusion of coils, persistence or even intensification of pain syndrome, radiation exposure to the doctor and patient, and allergic reactions to contrast agents and implanted coils [7,15,16]. Some authors suggest that protrusion of coils may be a cause of increased pelvic pain after EEGV [17,18]. Another reason of failure when using this technique can be the development of an allergic reaction to nitinol coils implanted into the gonadal veins [19,20]. The prevalence of allergy to nickel and other nickel-containing alloys in the general population is about 15% [21], and this factor should be taken into account when planning coil embolization for treating patients with PCS.

In the present study, we examined the incidence of PES after EEGV and the effect of venoactive drug therapy on its development and severity. The available data [10,22] and our own experience with MPFF [23] allowed authors to suggest that this drug is able to minimize EEGV-related complications. Previous studies have shown that MPFF reduces the PVP severity in patients with PCS [23].

In the present study, no difference was found between patients who received or did not receive MPFF before the intervention (18.4% vs. 28.1%, *p* = 0.58). Administration of VAD before and after endovascular intervention had no significant effect on the rate of PVP relief (81.6% and 71.8% in groups 1 and 2, respectively, *p* = 0.64). At the same time, group 1 patients reported less severity and shorter duration of PES: post-embolization pain score was 6.2 ± 0.4 vs. 7.8 ± 0.3 (*p* = 0.009), and duration of symptoms was three times shorter—5.0 ± 1.2 vs. 16.2 ± 2.7 days (*p* = 0.003). These data suggest that the use of MPFF in the perioperative period can reduce the severity and duration of PES and accelerate rehabilitation of patients with PCS who underwent EEGV with coils.

Coil protrusions were found in 4.2% of patients (all in the MPFF group). This fact suggests that treatment with MPFF cannot prevent coil protrusion. Their occurrence is probably associated with the technical peculiarities of EEGV that require using coils of 20% greater diameter than the calibre of target gonadal veins. This is necessary to prevent coil migration and to provide a reliable occlusion of the gonadal vein.

Coil protrusion cannot be detected with DUS, MSCT, or venography. This is not a venous wall perforation, but excessive invasion of coil whorls into the venous wall. This explains the absence of retroperitoneal hematoma and extravasation of contrast medium, according to radiological findings. In the present study, coil protrusion was suspected in some patients due to the presence of persistent (more than 1 month) pain in the area of gonadal vein embolization that did not respond to medications. Protrusions could be detected visually only during endoscopic resection of the left gonadal veins with implanted coils. It was found intraoperatively that retroperitoneal adipous tissue was scarce in all three patients. This can explain, at least partially, the cause of postprocedural pain. It could be probably triggered by mechanical contact of the swollen whorls of the coils with the left genitofemoral nerve. BMI of those women was significantly lower (mean 17.6 ± 0.5 kg/m^2^) compared to other patients. It should be noted that in all patients with PES, the BMI was lower than in the patients without PES (19.9 ± 0.2 kg/m^2^ and 24.3 ± 0.4 kg/m^2^, respectively).

These findings allowed us to hypothesize that patients with low body mass and lack of retroperitoneal fat tissue are more prone to the PES development and coil protrusion after EEGV. It cannot be excluded also that the rate of coil protrusion after EEGV in general practice is higher than the rate observed in our study. The sufficiently developed retroperitoneal tissue serves as a kind of damper protecting anatomical structures located near the gonadal veins (genitofemoral nerve, lumbar muscles). It is possible that inflammatory processes developing in the retroperitoneal tissue in the protrusion site subsides over time and under the influence of conservative treatment. A kind of fibrous cuff may develop around the vein, leading to absence of clinical manifestations of protrusion. This hypothesis is difficult to confirm in practice, as it can require removal of embolized gonadal veins in asymptomatic patients, which is an unacceptable intervention. Nevertheless, taking into account the data obtained, this assumption seems plausible. A sub-analysis of patients with coil protrusions irrespective to PES was based on the fact that PES was resolved after medical treatment, and the presence of protrusion required surgical intervention, after which the pain syndrome was completely eliminated.

Thrombosis of parametrial veins after EEGV was detected in 14 (20%) out of 70 patients. It was slightly more frequent in patients with PES and occurred more often in the patients after bilateral EEGV. While we found no difference in the rate of this condition between patients with and without PES, it may be assumed that development of thrombotic process in the visceral pelvic veins after EEGV may serve as one of the triggers of PES. Therefore, it seems reasonable to use anticoagulant prophylaxis of venous thromboembolism (VTE) after EEGV in patients with PCS.

We were unable to investigate immunological status of our patients due to the lack of data about allergic reactions to metals in medical history, so the role of allergic component in the pathogenesis of PES remains unclear. The occurrence of pain after EEGV was considered a local inflammatory response of the venous wall to implanted coils (Inconel^®^ nickel-chromium alloy). The use of non-steroidal anti-inflammatory drugs in combination with MPFF made it possible to achieve resolution of pain and hyperthermia without administration of corticosteroids and antihistamines, which may indirectly indicate a non-allergic nature of PES. At the same time, hypersensitivity to nickel in these patients could not be completely ruled out, taking into account that the alleged allergic reaction was mild, local, and sporadic. There were neither skin signs, such as discoloration, and papular rash, nor leg swelling after EEGV in patients with PES.

Prognosis for patients of both groups was good. We observed full disappearance of PES symptoms, including pain, in all the patients after one month. Nevertheless, duration of pain as well as its intensity in those who underwent EEGV led to prolonged time to return to normal activity and to work.

Our study has some limitations of which the most important was its open, non-randomized design; due to small number of patients that would have been available in practice for inclusion, we could not plan their enrollment at the beginning. It is also worth mentioning that we did not perform skin tests for metal allergy and assessments of the immunological status of patients.

## 5. Conclusions

Post-embolization syndrome is a frequent complication of the endovascular embolization of gonadal veins with coils. The treatment with VAD does not affect the rate of PES development, but significantly reduces its severity and duration and accelerates rehabilitation of patients after endovascular intervention. The study findings substantiate the use of VAD before and after EEGV in all patients with PCS. Low BMI and parametrial vein thrombosis should be considered possible predictors of PES development.

## Figures and Tables

**Table 1:** Baseline characteristics of groups and technical characteristics of interventions.

Parameter	Group 1, *n* = 38	Group 2, *n* = 32	*p* Value
Age, years	31.1 ± 2.2	30.4 ± 1.5	0.79
BMI, kg/m^2^	22.4 ± 0.6	23.6 ± 0.5	0.12
Disease duration, years	4.9 ± 1.7	5.4 ± 0.8	0.49
PVP, *n* (%)	38 (100)	32 (100)	-
PVP severity, scores (VAS)	7.8 ± 0.5	6.8 ± 0.3	0.09
Heaviness in the hypogastric region, *n* (%)	38 (100)	32 (100)	-
Dyspareunia, *n* (%)	30 (78.9)	21 (65.6)	0.28
Concomitant disorders with CPP, *n* (%)	0	0	-
Valve incompetence of LGV, *n* (%)	37 (97.3)	30 (93.7)	0.58
Valve incompetence of RGV, *n* (%)	6 (15.8)	4 (12.5)	0.74
LGV diameter, mm	8.1 ± 0.3	7.7 ± 0.6	0.55
RGV diameter, mm	6.8 ± 0.4	6.5 ± 0.3	0.55
Side of embolization			
Left-sided, *n* (%)	32 (84.2)	28 (87.5)	0.52
Right-sided, *n* (%)	1 (2.6)	2 (6.2)	0.58
Bilateral, *n* (%)	5 (13,1)	2 (6.1)	0.56
Number of implanted coils, n			
Left-sided, *n*	5.2 ± 0.5	4.8 ± 0.6	0.61
Right-sided, *n*	3.4 ± 0.3	3.5 ± 0.6	0.88
Bilateral, *n*	9.1 ± 0.7	8.8 ± 1.2	0.82
PV thrombosis after EEGV, *n* (%)	8 (21.05)	6 (18.7)	0.48

Abbreviations: BMI, body mass index; CPP, chronic pelvic pain; EEGV, endovascular embolization of the gonadal veins; LGV, left gonadal vein; PV, parametrial veins; PVP, pelvic venous pain; RGV, right gonadal vein; VAS, visual analogue scale.

**Table 2:** Main results of the study and comparison of groups.

Parameter	Total, *n* = 70	Group 1, *n* = 38	Group 2, *n* = 32	*p* Value
Post-embolization syndrome, *n* (%)	16 (22.9%)	7 (18.4%)	9 (28.1%)	0.40
PVP relief, *n* (%)	54 (77.1%)	31 (81.6%)	23 (71.8%)	0.40
Coil protrusions, *n* (%)	3 (4.3%)	3 (4.3%)	0	-
Post-embolization pain (VAS score)	-	6.2 ± 0.4 *	7.8 ± 0.3	0.009
Duration of PES, days	-	5.0 ± 1.2 *	16.2 ± 2.7	0.003
Thrombosis of parametrial veins, *n* (%)	14 (21.2%) *	8 (23.5%) *	6 (18.9%)	1.0

* Excluding 3 patients from group 1 who had undergone resection of embolized gonadal veins (*n* = 35).

**Table 3:** Comparison between patients with and without PES development (*n* = 70).

		Patients with PES, *n* = 16	Patients without PES, *n* = 54	*p* Value
PVP before embolization, VAS scores		7.3 ± 0.4	7.5 ± 0.5	0.75
PVP after embolization, VAS scores		8.2 ± 0.2	5.1 ± 0.3	0.0001
BMI, kg/m^2^		19.9 ± 0.2	24.3 ± 0.4	0.0001
Diameter of the gonadal veins				
Left gonadal vein, mm		7.3± 0.3	7.6 ± 0.4	0.55
Right gonadal vein, mm		6.5 ± 0.4	6.6 ± 0.3	0.84
Side of embolization	Left, *n* (%)	14	49	0.28
	Right, *n* (%)	0	3	
	Bilateral, *n* (%)	2	2	
Number of coils	Left-sided, *n*	5.8 ± 0.4	5.5 ± 0.3	0.76
	Right-sided, *n*	0	4.0 ± 0.4	
	Bilateral, *n*	9	9.4 ± 0.3	
Thrombosis of parametrial veins, *n* (%)		4 (25%)	10 (18.5%)	0.82
